# Is Rescuer Cardiopulmonary Resuscitation Jeopardised by Previous Fatiguing Exercise?

**DOI:** 10.3390/ijerph17186668

**Published:** 2020-09-13

**Authors:** J. Arturo Abraldes, Ricardo J. Fernandes, Núria Rodríguez, Ana Sousa

**Affiliations:** 1 Department of Physical Activity and Sport, Faculty of Sports Sciences, University of Murcia, 30720 Murcia, Spain; abraldes@um.es; 2 Porto Biomechanics Laboratory, University of Porto, 4200-450 Porto, Portugal; 3 Centre of Research, Education, Innovation and Intervention in Sport, Faculty of Sport, University of Porto, 4200-450 Porto, Portugal; 4 Department of Physical Activity and Sport, Catholic University of San Antonio, 30107 Murcia, Spain; nuriarodriguezsuarez@gmail.com; 5 Research Center for Sports, Exercise and Human Development, 5001-801 Vila Real, Portugal; sousa.acm@gmail.com; 6 University Institute of Maia, 4475-690 Maia, Portugal

**Keywords:** physiology, fatigue, cardiopulmonary resuscitation, oxygen uptake

## Abstract

Survival outcomes increase significantly when cardiopulmonary resuscitation (CPR) is provided correctly, but rescuer’s fatigue can compromise CPR delivery. We investigated the effect of a 100-m maximal run on CPR and physiological variables in 14 emergency medical technicians (age 29.2 ± 5.8 years, height 171.2 ± 1.1 cm and weight 73.4 ± 13.1 kg). Using an adult manikin and a compression-ventilation ratio of 30:2, participants performed 4-min CPR after 4-min baseline conditions (CPR) and 4-min CPR after a 100-m maximal run carrying emergency material (CPR-run). Physiological variables were continuously measured during baseline and CPR conditions using a portable gas analyzer (K4b^2^, Cosmed, Rome, Italy) and analyzed using two HD video cameras (Sony, HDR PJ30VE, Japan). Higher VO_2_ (14.4 ± 2.1 and 22.0 ± 2.5 mL·kg^−1^·min^−1^) and heart rate (123 ± 17 and 148 ± 17 bpm) were found for CPR-run. However, the compression rate was also higher during the CPR-run (373 ± 51 vs. 340 ± 49) and between every three complete cycles (81 ± 9 vs. 74 ± 14, 99 ± 14 vs. 90 ± 10, 99 ± 10 vs. 90 ± 10, and, 101 ± 15 vs. 94 ± 11, for cycle 3, 6, 9 and 12, respectively). Fatigue induced by the 100-m maximal run had a strong impact on physiological variables, but a mild impact on CPR emergency medical technicians’ performance.

## 1. Introduction

High quality chest compressions are crucial for successful cardiopulmonary resuscitation (CPR), and poor application can compromise the emergency situation outcome [1]. Several studies have indicated a significant relationship between survival outcomes and CPR quality, particularly chest compression depth [2,3], rate [4] and fraction [5]. The current guidelines (the American Heart Association (AHA) and the European Resuscitation Council (ERC)) describe standard CPR as administrated using a single approach for all adult patients—30:2 compress/ventilation ratio [6,7]—with chest compressions delivered at a rate and depth of at least 100 to 120 compressions/min and 5 cm (but no more than 6 cm), allowing full chest recoil between compressions and minimizing interruptions [6]. However, there are several physiological constraints that can significantly affect application in accordance with current guidelines and, therefore, compromise the CPR result [8].

The delivery of high-quality manual chest compressions is challenging. Rescuer’s fatigue is a likely contributor to variability of application and, thus, inflation quality during resuscitation [1]. CPR after a life-threatening emergency is sometimes performed with the technician in a non-resting state, since he/she might perform an intense run from the emergency vehicle to the person in need. This can lead to rescuer fatigue and chest compressions depth can degrade after 1 min of CPR delivery [9] (degrading continuously up to 3 min [10]). Rescuers themselves are not able to recognize when fatigue begins to affect their performance [11]. Furthermore, it is known that patient’s survival following CPR after cardiac arrest depends on adequate myocardial blood flow [12,13], which is more adequately provided when end tidal carbon dioxide (PetCO_2_) is monitored [14]. In fact, PetCO_2_ serves as a non-invasive measurement of blood flow generated by chest compression during resuscitation [15]. Low PetCO_2_ values during CPR have been associated with decreased return of spontaneous circulation rates and increased mortality, and high PetCO_2_ values related with a better return of spontaneous circulation rates and survival [16,17]. Optimal values of this variable depend on chest compression quality, ventilation rate and tidal volume [18], all of which might deteriorate with rescuer fatigue.

Although a direct relationship between survival outcomes and CPR quality variables has been shown, most studies analyzed rescuer’s fatigue during the specific CPR application period. Nevertheless, information on the effect of preceding strenuous exercise on the quality of chest compressions is limited. It is still unclear if high-quality inflation and chest compression is influenced by fatigue experienced in a typical CPR emergency setting. These findings can provide knowledge leading to better training and education for those providing CPR and, therefore, improve survival outcomes. Our purpose was to analyze the effect of a fatigue inducing 100 m maximal run with equipment on CPR performance and cardiorespiratory demand. It was hypothesized that previous exercise would induce a higher CPR physiological demand, even in well trained personnel.

## 2. Materials and Methods

### 2.1. Subjects

Fourteen (nine males and five females) CPR trained (4–6 h in 2–3 weekly training sessions) and moderately active certified emergency medical technicians participated in the current study. They obtained their diploma from the Spanish Lifesaving Royal Federation, which follows the study plan of the International Lifesaving Federation. Their main characteristics were: 29.6 ± 6.3 vs. 28.6 ± 5.7 years, 177.1 ± 4.7 vs. 160.1 ± 13.6 cm and 78.6 ± 10.5 vs. 64.1 ± 12.9 kg for male and female (respectively). Individual written informed consent was provided, and participants were familiarized with the testing procedures during a pre-experiment session. The study was conducted in accordance with the Declaration of Helsinki and journal guidelines. The ethics committee of the local institution approved the study design (Lifesaving Federation of Galicia code no. 2352/2015).

### 2.2. Design

**Figure 1:**
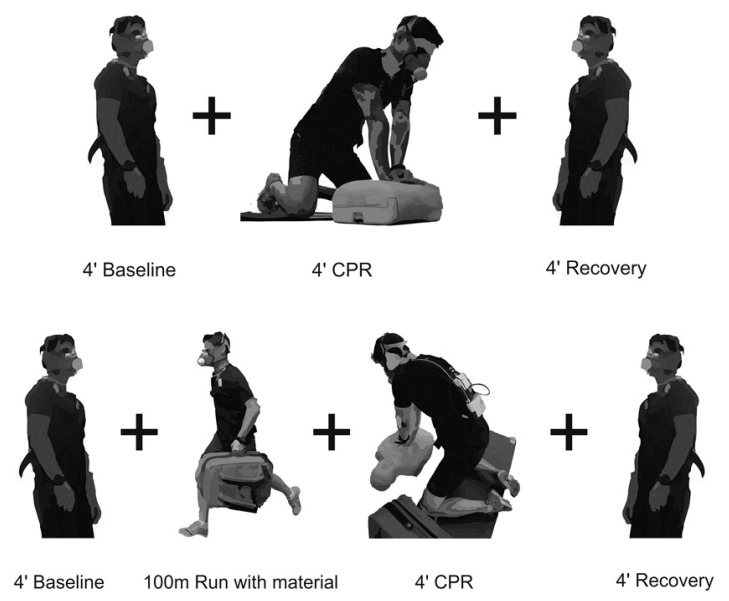
Testing protocols: (i) 4-min baseline + 4-min cardiopulmonary resuscitation (CPR) + 4-min recovery (upper panel) and (ii) 4-min baseline + 100-m Run with the material + 4-min CPR + 4-min recovery (lower panel). The manikin used is in accordance with the American Heart Association (AHA) and European Resuscitation Council (ERC) guidelines for CPR practice.

After standing upright for 4 min to assess baseline physiological variables, participants performed a 4 min mouth-to-mouth CPR on an adult manikin (Laerdal^®^ Resusci Anne Torso; Laerdal Medical, Stavanger, Norway) with a 30:2 compression-ventilation ratio and 5 cm compression depth (in accordance with stablished guidelines [7,19]. At the end of the 4 min CPR, participants reassumed the upright position to recover for 4 min or until the baseline physiological values were reached. Then, participants performed a 100 m straight run (± 2° decline) at maximal intensity carrying standard emergency material (~10 kg) followed by 4 min mouth-to-mouth CPR, and a 4 min recovery period (as previously described; see Figure 1: CPR-run). In both experimental procedures, due to the fact that the participant was wearing the physiological testing mask, the two mouth-to-mouth ventilations were simulated, on the manikin after each 30-compression cycle.

### 2.3. Methodology

Gas exchange variables were continuously measured breath-by-breath during baseline, CPR and recovery periods using a telemetric portable gas analyzer (K4b^2^, Cosmed, Rome, Italy) placed near the rescuers center of mass, adding only 800 g to their total weight (for a detailed analysis of this apparatus and its use in different exercise mode settings see [20,21]. The gas analyzer and the turbine volume transducer were calibrated before each test with gases of known concentration (16% O_2_ and 5% CO_2_) and a 3 L syringe, respectively. Heart rate (HR) was monitored continuously with a Polar Vantage NV (Polar electro Oy, Kempele, Finland) that telemetrically transferred data to the K4b^2^ portable unit. In addition, in both tests, the 4 min CPR procedure was recorded using two HD video cameras (Sony, HDR PJ30VE, Japan; 100 Hz frequency) placed frontal and laterally on rigid tripods (HAMA Star 63, Spain) at 1 m height and 3 m distance from the participant to assure the procedure quality. The temporal analysis of the CPR techniques was done manual and independently by two experts, using Media Player Classic Home Cinema (MPC-HC v.1.7.10. 64 bits, Microsoft Windows, Microsoft Corporation, Albuquerque, NM, USA) with a 0.96 intra-test correlation.

Initially, errant breaths (e.g., coughing and signal interruptions), which do not truly represent the physiological functioning during exercise, were omitted from the oxygen uptake (VO_2_) analysis by including only those that were between mean ± four standard deviations [22]. Afterwards, individual breath-by-breath VO_2_ responses were smoothed using a three-breath moving average and time averaged every 10s [22]. Respiratory frequency (RF), tidal volume (TV), minute ventilation (VE), VO_2_, volume of carbon dioxide expired (VCO_2_), HR, respiratory quotient (R) and PetCO_2_ were measured throughout the 4 min baseline (4 min average) and the 4 min in both CPR tests (4 min average and average over each min). The total number of compressions were determined in both 4 min CPR conditions and the compression rate was measured over the first, second, third and fourth three complete CPR cycles (compressions 1–90, 91–180, 181–270 and 271–360, respectively). The correct depth of the compressions (5 cm) was controlled by an acoustic signal placed on the adult manikin. Therefore, for analysis purposes, the compressions that induced an acoustic signal were considered “correct” and the remaining the “incorrect”. The task intensity was rated after all CPR conditions using the Borg scale (RPE) [23].

### 2.4. Statistical Analysis

All physiological and CPR related variables were checked for normality and homogeneity using the Shapiro-Wilk test, and reported as mean ± SD. Differences between CPR protocols were tested using a paired t-test and, when different cycles were analyzed within the same condition. An ANOVA for repeated measures was conducted (significant effects were further explored using Bonferroni post hoc procedures). Pearson product-moment correlation coefficient was used to test the relationships between all variables. Magnitudes of standardized effects |f| were determined against the following criteria: small (0.2–0.5), moderate (0.5–0.8) and large (>0.8). All statistical procedures were conducted with SPSS 21.0 (IBM, Armonk, NY, USA) and statistical significance was set at *p* < 0.05.

## 3. Results

**Figure 2:**
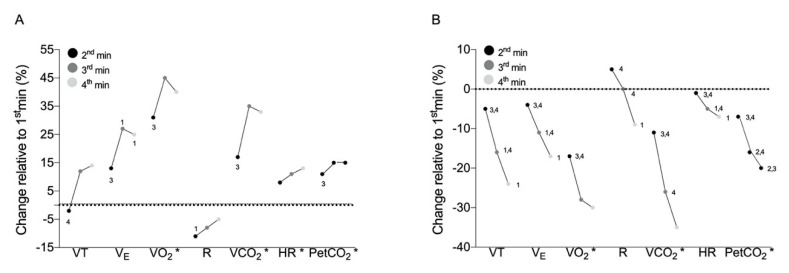
Percentage of change of the cardiopulmonary variables in the 2nd, 3rd and 4th min relative to the 1st min in CPR (**A**) and CPR-Run (**B**) conditions (the horizontal dashed line represents the 1st-min mean value). 1,2,3,4 Different from the 1st, 2nd, 3rd and 4th min, respectively; * 1st-min different from all min (*p* < 0.05).

The fatigue inducing 100 m run lasted 28 ± 8 s, with Table 1 showing the values of the cardiopulmonary variables assessed during baseline and over each min of the CPR tests. Apart from RF (in both 2nd and 3rd min) and PetCO_2_ (in the 3rd min), the 100 m run produced higher overall values for all physiological variables (*p* < 0.001). In addition, higher values (*p* < 0.001) were observed for all variables over the 4 min during CPR-run compared with the CPR condition (except for PetCO_2_). Concomitantly CPR-run presented higher RPE values compared with CPR (5.2 ± 1.6 vs. 3.1 ± 0.9 a.u. in a scale of 0 to 10 units; *p* < 0.001). The percentage of change relative to the 1st min in both CPR and CPR-run conditions is presented in Figure 2.

In the CPR condition, VO_2_, VCO_2_, HR and PetCO_2_-1st min were lower from the 2nd min (*p* ≤ 0.02; η^2^ = 0.62, 0.51, 0.22 and 0.88, respectively), and VE, VO_2_, VCO_2_ and PetCO_2_-2nd min were lower compared to the 3rd min (*p* ≤ 0.03). TV and R-2nd min were lower than the 1st and 4th min (*p* ≤ 0.02), and VE-3rd min was higher and -4th min lower compared to the -1st min (*p* ≤ 0.01). In CPR-run, VO_2_, VCO_2_ and PetCO_2_-1st min were higher compared to the 2nd min (*p* < 0.001; η^2^ = 0.77, 0.86 and 0.89), and TV, VE, VO_2_, VCO_2_, HR and PetCO_2_ evidenced a similar behavior since the 2nd min was higher compared with the 3rd and 4th min (*p* ≤ 0.02). TV, VE and HR were lower at the 3rd comparing to the 1st min (*p* ≤ 0.01), but higher than the 4th min (*p* ≤ 0.03). Both VO_2_ and HR-2nd min were lower than the 1st min (*p* ≤ 0.04), but higher than the 3rd and 4th min (*p* ≤ 0.02). VO_2_ and HR-4th min were also lower compared with the 1st min (*p* < 0.001). Both R-2nd and 3rd min were higher than the 4th min (*p* < 0.001), with this latter value being lower compared with the 1st min (*p* < 0.03). PetCO_2_ mean values were different in-between min and RF values were similar over each min in both CPR and CPR-run conditions.

**Figure 3:**
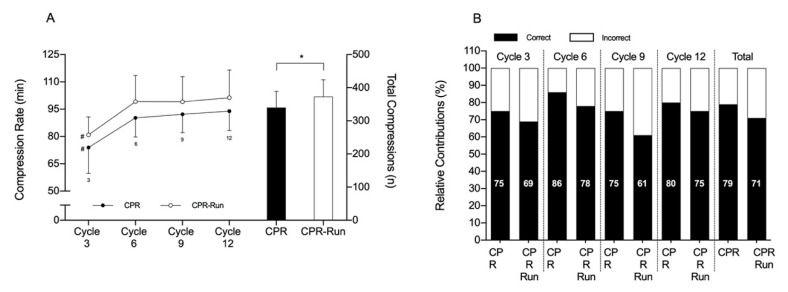
Mean ± SD values for compression rate and total number of compressions (**A**), and mean relative values for correct and incorrect compressions (**B**) over the first, second, third and fourth (cycle three, six, nine and 12, respectively) three complete CPR cycles, when preceded by 4-min baseline (CPR) and 100 m maximal run (CPR-Run), conditions; three, six, nine, 12 different from cycles three, six, nine and 12 of the CPR-Run condition, respectively; # different from cycles six, nine and 12 within the same condition; * differences between CPR and CPR-Run condition (*p* < 0.05).

The compression rate and correct/incorrect compressions assessed from the first to the last complete three CPR cycles, as well as the total number of compressions in both tests, are displayed in Figure 3. The compression frequency was higher in the CPR-run than in the CPR condition (373 ± 51 vs. 340 ± 49; *p* = 0.004), but values were highly correlated (r = 0.85; *p* = 0.002). For all cycles, the compression rate was also greater for CPR-run compared to CPR (80.9 ± 9.8 vs. 73.9 ± 14.2, 99.4 ± 14.3 vs. 90.2 ± 10.5, 99.1 ± 13.2 vs. 92.2 ± 10.1 and 101.2 ± 15.2 vs. 93.9 ± 10.6 for cycles three, six and nine and 12; *p* ≤ 0.02). In addition, the compression rate from the first to the remaining subsequent three CPR complete cycles were lower in both conditions (*p* < 0.001, η^2^ = 0.84 and 0.83 for CPR and CPR-run). No differences were found between correct and incorrect compressions in either condition.

## 4. Discussion

The purpose of the current study was to analyze the effect of fatigue on CPR and cardiopulmonary variables in certified emergency medical technicians. Our hypothesis was partially verified since the 100 m maximal run had a strong physiological impact during CPR. However, previous fatiguing exercise had no evident influence on CPR compression rate and depth (comparing to the values obtained after a 4 min resting period). Thus, even if the 100 m maximal run performed by the rescuers was not up to the level of elite runners, medical personnel seem to be sufficiently well prepared for doing it fast and maintain the CPR quality when in a fatigue state.

Recently, the European Resuscitation Council has recommended a TV range between 500 to 600 mL [6], a variable closely associated with peak airway pressure during CPR. In fact, managing peak airway pressure is fundamental to avoid stomach inflation and, by reducing TV, controlling peak flow rate [24]. In the current study, TV values over the 4 min CPR and CPR-run were 1360 and 2270 mL (ranging from 1300 to 1480 mL and 2000 to 2600 mL in the 1st and 4th min, respectively). TV was assessed through participants expired air (using a facemask), with the results being three to four-fold higher than the recommended ones. This suggests that they were high enough to ensure correct ventilation in the simulated CPR. In fact, this excess in ventilation volume and flow rate were recently reported in two studies conducted with lifeguards [18,22].

Lower TV (~500 vs. 1000 mL) in adult patients with respiratory arrest maintains good oxygenation and carbon dioxide elimination (while decreasing peak airway pressure), making stomach inflation less likely [24]. In the current study, TV was higher in CPR-run compared with CPR and, although the time necessary to run the 100 m to reach the manikin was short (<30 s), it induced a higher cardiopulmonary response clearly visible along the 4 min CPR. This was also reflected by the higher RPE values reported in the CPR-run condition (~3 vs. 5 a.u.), as well as by the ~27 and 30 breaths/min of RF (or ventilation rate) for CPR and CPR-run. By extrapolating from compression rate values (~85 and 93 per min for CPR and CPR-run), it was shown that our participants performed ~6 breaths/min in the manikin, less than the 10 to 12 breaths/min suggested in international resuscitation guidelines [6]. However, no relevant differences in ventilation rate between survivors and non-survivors was previously reported [25]. As was the case for TV, RF was also higher in CPR-run compared to the CPR condition.

Complementarily to the high quality chest compressions and ventilation, PetCO_2_ is a fundamental CPR survival outcome, evaluating cardiac output at low-flow rates and helping assess CPR effectiveness [6]. Of the evaluated cardiopulmonary outcomes, only PetCO_2_ showed a similar behaviour in the overall 4 min CPR and CPR-run (~33 vs. 32 mmHg). This ranged from ~24 to 27 mmHg in patients that had in-hospital and out-of-hospital cardiac arrest and is independent of compression rate [26]. When ventilation is held constant, ideally in both rate and tidal volume, PetCO_2_ becomes an excellent measure of pulmonary blood flow [13]. In the current study, even if not directly measured in the manikin (but through the rescuers expired air), PetCO_2_ mean values found suggest that a CPR of adequate quality was provided. In support of our results, a cut point of 25.5 mmHg was established for initial PetCO_2_ [15]. For patients with initial PetCO_2_ < 25.5 mm Hg, survival benefit ceased at an earlier point in resuscitation, whereas above this threshold, the probability of survival cumulatively increased for a longer period. Optimal PetCO_2_ depend also, among other factors, on chest compression quality, ventilation rate and tidal volume [18] all which deteriorate with fatigue and, although a similar behavior was observed in the overall 4 min CPR, it was higher in the 1st, 2nd and 4th min of the CPR-run.

The influence of fatigue on CPR performance is shown in Figure 2. The change (compared to the 1st min) in the CPR outcomes showed an increase of ~32% on average (ranging 5 to 45%) for all cardiopulmonary variables (except for TV-2nd min and R). In contrast, all cardiopulmonary variables decreased ~15% (from 2 to 38%) along the CPR-run (except the R-2nd min), indicating physiological fatigue induced by the previous 100 m maximal run (as previously suggested [21]). In addition, the mean R value, a well-accepted indicator of exercise intensity, was higher in the CPR-run than in the CPR condition (1.25 vs. 0.92, ranging from 1.27 to 1.16 and 0.97 to 0.92, respectively), also suggesting that a previous intensive exercise leads to a relevant physiological fatigue during CPR.

In the current study, the mean VO_2_ and HR in the CPR condition represented only a moderate physiological demand (14.4 mL·kg^−1^·min^−1^ and 123 bpm, ranging between 11.4–16.5 mL·kg^−1^·min^−1^ and between 115–130 bpm). Similar metabolic demands were previously reported [27,28]. However, emergency medical technicians setting mostly involve being at rest, but with the occasional requirement to handle CPR in a limited time period after running at a high intensity to get to the victim as quickly as possible. The mean cardiovascular demands associated with performing this specific task were significantly higher (22 mL·kg^−1^·min^−1^ and 142 bpm, ranging between 13.3–27.2 mL·kg^−1^·min^−1^ and between 144–155 bpm) and approximately equivalent to those seen at continuous moderate intensity (60–70% of maximum intensity) in a range of different exercise modes [20]. In healthy subjects, this is the highest work percentage recommended to avoid excessive anaerobic metabolism and fatigue. Considering that the energetic demands placed on the rescuer depend upon the specific role assumed (e.g., performing on a flat surface, steep slope, stairs), rescuers occupational health training should include a variety of different exercises covering potential incident situations.

International resuscitation guidelines recommend that chest compressions should be delivered at a rate of, at least, 100 to 120 compressions/min [6], with a prompt CPR delivery having an essential role in the survival chain for cardiac arrest resuscitation. In fact, a recent work has demonstrated that return of spontaneous circulation from in-hospital cardiac arrest was associated with higher chest compression rates, consistent with out-of-hospital studies of CPR quality [29,30]. However, in the current study, the number of compressions/min were slightly lower than that suggested (~85 and 93 compressions/min for CPR and CPR-run), which is in line with six independent studies reporting that survivors have received 85 to 100 compressions/min chest compression rates, compared with non-survivors who received a lower rate [25]. In the current study, the compression rate over the first three complete cycles was lower than the subsequent three CPR complete cycles for both CPR and CPR-run (~74 and 80 compressions/min), underestimating the total number of compressions assessed over the 4 min (373 vs. 340 compressions/min). In fact, the second, third and fourth three complete CPR cycles (cycle three, six, nine and 12, respectively), in both conditions, were very close to those recommend from international resuscitation guidelines [6].

Unexpectedly, but in line with a previous 100 m simulated in-water rescue study [21], the mechanical related fatigue did not decisively influence rescuers CPR-run performance. In fact, the compression rate was higher for CPR-Run compared with the non-fatigue state corroborating previous findings that after a simulated water-rescue the rate of compressions increased compared to a resting control setting [31]. We have tried to upgrade Sousa et al. [21] results by analyzing characterizing in more detail the fatigue-induced deterioration over the four complete CPR cycles, focusing on the number of correct/incorrect compressions (defined as 30 and <30, respectively). In the literature [9,11], compressions were judged as correct if both depth and placement were in accordance with standard advanced cardiac life support guidelines. The % of correct and incorrect compressions, as well as the total number of compressions per min, remained similar from the 1st to the 5th (last) min of CPR. High-quality chest compressions are a key determinant of patient survival, although the sustained delivery of high-quality CPR is infrequently achieved in clinical practice.

Notwithstanding, it has been suggested that values equal to or greater than 70% effective compression and ventilation will deliver, CPR of sufficient quality [32]. The mean relative values for correct compressions in CPR condition was always greater than 70% (79% in average). However, in the CPR-run condition this value was 71%, when in cycle 1 was achieved 69% with the lowest value in cycle nine (61%). Therefore, our results show that the fatigue caused by the execution of the 100 m maximal run had some impact on quality, at least, in the beginning and near the end of the 4 min CPR. This may be due to anthropometric differences and the physical capacities of the rescuers. It was suggested that physical fatigue affects males less and with a BMI ≤ 25 kg·m^2^ [33]. From the 14 participants of the current study, five were females and with a BMI of 25 kg·m^2^, which could help explain the confounding results. Training could also be a variable that influences results and would be a key point in the good execution of CPR. The present results seem to suggest that, and although considering the absence of differences between correct and incorrect compressions, as well as the higher number of compressions in each three complete CPR cycles and over the 4 min CPR, previous fatiguing exercise might have jeopardized the quality of chest compressions.

## 5. Conclusions

The 100 m maximal run induce a relevant physiological stress over each min and in the overall 4 min CPR compared with starting a CPR from a resting state. The previous induced fatigue was not reflected in the quality of CPR since the total number of compressions and the compression rate from the first and three subsequent complete cycles were higher in CPR-run than in CPR. However, previous fatiguing exercise might have jeopardized the quality of chest compressions, as this was lower than 70% correct compressions during half of the 4-min CPR duration. This physiological constraint should be taken into consideration during the training process of certified rescuers, helping them to deal with this in emergency situations. Meanwhile, although the CPR was performed direct on the manikin, the ventilations were not (due to the breathing facemask worn by the rescuers). Therefore, in future studies, it will be important to use the new generation devices (that are already available) to measure rescuers ventilatory variables while performing CPR in line with European Resuscitation Council guidelines.

## Figures and Tables

**Table 1:** Mean ± SD values for the cardio-pulmonary variables assessed during baseline (base) and over each minute and over the 4-min in sessions 1 (CPR) and 2 (CPR-Run).

Variables		Rf (b.min^−1^)	VT (l)	V_E_ (l.min^−1^)	VO_2_ (ml/kg/min)	R	VCO_2_ (ml.min^−1^)	HR (bpm)	PetCO_2_ mmHg
CPR	Base	15.4 ± 3.7	1.1 ± 0.3	15.3 ± 3.5	6.2 ± 1.1	0.88 ± 0.1	0.4 ± 0.1	90 ± 12	34.4 ± 2.5
	1st min	26.3 ± 8.9 ^1^	1.30 ± 0.6 ^1^	30.1 ± 8.4 ^1^	11.4 ± 1.8 ^1^	0.97 ± 0.0 ^1^	0.81 ± 0.2 ^1^	115 ± 18 ^1^	28.7 ± 4.4 ^1^
	2nd min	29.6 ± 9.8	1.27 ± 0.4 ^2^	33.9 ± 7.4 ^2^	14.9 ± 1.9 ^2^	0.87 ± 0.1 ^2^	0.95 ± 0.2 ^2^	124 ± 15 ^2^	30.8 ± 5.2 ^2^
	3rd min	28.5 ± 7.7	1.46 ± 0.4 ^3^	38.3 ± 7.7 ^3^	16.5 ± 2.7 ^3^	0.90 ± 0.0 ^3^	1.09 ± 0.2 ^3^	128 ± 15 ^3^	33.1 ± 4.6
	4th min	27.1 ± 7.2 ^4^	1.48 ± 0.4 ^4^	37.7 ± 9.3 ^4^	16.0 ± 3.2 ^4^	0.92 ± 0.1 ^4^	1.08 ± 0.2 ^4^	130 ± 16 ^4^	31.4 ± 4.4 ^4^
	4-min	27.1 ± 8.3 *	1.36 ± 0.4 *	33.9 ± 7.3 *	14.4 ± 2.1 *	0.92 ± 0.1 *	0.96 ± 0.2 *	123 ± 17 *	32.9 ± 5.0
CPR Run	Base	14.7 ± 1.2	1.1 ± 0.3	14.8 ± 2.5	7.7 ± 1.6	0.89 ± 0.1	0.4 ± 0.1	85 ± 10	33.7 ± 2.1
	1st min	29.9 ± 5.8	2.6 ± 0.8	74.9 ± 17.3	27.2 ± 3.0	1.27 ± 0.1	2.51 ± 0.5	155 ± 15	39.6 ± 5.3
	2nd min	30.3 ± 5.6	2.48 ± 0.7	72.4 ± 17.7	22.8 ± 3.2	1.33 ± 0.1	2.24 ± 0.5	154 ± 13	37.2 ± 4.9
	3rd min	31.1 ± 5.7	2.20 ± 0.7	66.7 ± 16.7	19.8 ± 2.6	1.27 ± 0.1	1.86 ± 0.4	148 ± 15	33.7 ± 4.9
	4th min	32.1 ± 5.5	2.0 ± 0.5	62.6 ± 14.7	19.3 ± 2.9	1.16 ± 0.1	1.65 ± 0.3	144 ± 15	35.6 ± 5.4
	4-min	30.5 ± 5.8	2.27 ± 0.6	67.3 ± 15.0	22.0 ± 2.5	1.25 ± 0.1	2.01 ± 0.4	148 ± 17	31.9 ± 5.3

Rf: respiratory frequency; VT: tidal volume; VE: minute ventilation; VO_2_: volume of oxygen consumed; R: respiratory quotient; VCO_2_: volume of carbon dioxide expired; HR: heart rate; PetCO_2_: tidal carbon dioxide; ^1^, ^2^, ^3^, ^4^ significant different from the 1st, 2nd, 3rd and 4th minute of the CPR-Run condition, respectively; * significant different from CPR-Run condition.
